# Supplementary material for: Chromothripsis during telomere crisis is independent of NHEJ, and consistent with a replicative origin
Source: Genome Res. 2019 May;29(5):737–49. doi: 10.1101/gr.240705.118 (PMC6499312; doi:10.1101/gr.240705.118)
Supplement: Supplemental Material [file supp_gr.240705.118_Supplemental_file_1.zip › contigs/annotated_contigs/DB84/contig.2.DB84_length_677_mean_cov_8.64106351551.docx]

**DB84_length_677_mean_cov_8.64106351551**

```
AGAGTGATCTTTTCTCAGGGCCATCCCCCTCCATTTTGTTCCTATTCTTTCTAATTCCTTCAAAAAAATCAGATCAGCTTTTCTTTCAA
 >chr6:19370603-19370986 - E=1e-218
GACTCAAGTAAATTTTAAAAAGTCAGGATTGATTTCTTACTGTGAACAAATTGAGGCATCAAAGTAAAGTTAAGAGGTTCCTTCCCACT

CTGTTCTTCTGCGGCTGCTCTGGTCGTATACATTTGAGGACGGTAAATATTATCATGGAGACTTAGCTGTGTTATTATAAGCTTACATT

TTACATATGCTGATTTGGGAGGACCTAAATTTACTACTGTAACATGGCATCACCTCTGGCTTTTACACACAGAGAGTGGCACAATTTTC

TTCCATTTTCTTCATGTATATGTTGA|A|TTTTTATTTGCTGTGAGAAAATAATTTGTATGCACCAATAAAAGTAGGAACTCCCTTTAC
                           >chr6:19369176-19369471 - E=1e-165
CCTGACCTTTTGCCTGACTTTAGGTTGGCTGAACAATTGACCATATCTCTCCCATTCTCTTTATAAGGAGAAGAGGAAGAAATGTCTTC

CCATTTTGGCACACAGAACACCAGGAAGAAATGTGTTTAACCCATACAAAGCACACTCCATTGATATGTAGTATATCTCTGTTCCCAGT

TGCTCTACTTGTGGGATTCTCTTCTCTTGACCTTTGCTCTAATAACAGAAGTGTGC
```
